# The impact of disease changes and mental health illness on readapted return to work after repeated sick leaves among Brazilian public university employees

**DOI:** 10.3389/fpubh.2022.1026053

**Published:** 2023-01-09

**Authors:** Adriano Dias, Hélio Rubens de Carvalho Nunes, Carlos Ruiz-Frutos, Juan Gómez-Salgado, Melissa Spröesser Alonso, João Marcos Bernardes, Juan Jesús García-Iglesias, Juan Ramón Lacalle-Remigio

**Affiliations:** ^1^Department of Public Health, Botucatu Medical School, São Paulo State University (UNESP), Botucatu, Brazil; ^2^Public/Collective Health Graduate Program, Botucatu Medical School, São Paulo State University (UNESP), Botucatu, Brazil; ^3^Graduate Program in Nursing Academic Master's and Doctoral Programs, Botucatu Medical School, São Paulo State University (UNESP), Botucatu, Brazil; ^4^Department of Sociology, Social Work and Public Health, Faculty of Labour Sciences, University of Huelva, Huelva, Spain; ^5^Safety and Health Postgraduate Programme, Universidad Espíritu Santo, Guayaquil, Ecuador; ^6^Department of Preventive Medicine and Public Health, Faculty of Medicine, University of Sevilla, Sevilla, Spain

**Keywords:** absenteeism, return to work, readaptation, Targeted Machine Learning, logistic regression

## Abstract

**Introduction:**

Health affects work absenteeism and productivity of workers, making it a relevant marker of an individual's professional development.

**Objectives:**

The aims of this article were to investigate whether changes in the main cause of the sick leaves and the presence of mental health illnesses are associated with return to work with readaptation.

**Materials and methods:**

A historical cohort study was carried out with non-work-related illnesses suffered by statutory workers of university campuses in a medium-sized city in the state of São Paulo, Brazil. Two exposures were measured: (a) changes, throughout medical examinations, in the International Classification of Diseases (ICD-10) chapter regarding the main condition for the sick leave; and (b) having at least one episode of sick leave due to mental illness, with or without change in the ICD-10 chapter over the follow-up period. The outcome was defined as return to work with adapted conditions. The causal model was established *a priori* and tested using a multiple logistic regression (MLR) model considering the effects of several confounding factors, and then compared with the same estimators obtained using Targeted Machine Learning.

**Results:**

Among workers in adapted conditions, 64% were health professionals, 34% had had changes in the ICD-10 chapter throughout the series of sick leaves, and 62% had diagnoses of mental health issues. In addition, they worked for less time at the university and were absent for longer periods. Having had a change in the illness condition reduced the chance of returning to work in another function by more than 30%, whereas having had at least one absence because of a cause related to mental and behavioral disorders more than doubled the chance of not returning to work in the same activity as before.

**Conclusion:**

These results were independent of the analysis technique used, which allows concluding that there were no advantages in the use of targeted maximum likelihood estimation (TMLE), given its difficulties in access, use, and assumptions.

## 1. Introduction

Absenteeism is a relevant marker of the workers' life cycle. In 2020, rates of work sickness absence for public and private sector workers in the UK were 2.7% and 1.6%, respectively, and thus statistically higher for public sector employees (1). In terms of health economics, absence from work leads to a high economic burden to society in terms of indirect costs due lost productivity (also called absenteeism costs) (2). On the other hand, from an occupational health and safety perspective, work absences represent problems that stem from working conditions, either due to precarious and unsafe relationships (3) or to changes in the environment (4), when they are caused by occupational injuries or illnesses. It is important to note, however, that a large proportion of sickness absences are due to non-work-related injuries and illnesses (i.e., injuries and diseases of non-occupational origin) (5).

Although some cases of sickness absence are episodical, there are others which are repeated. Repeated sick leave episodes may be quite frequent, a recent study with Brazilian public workers has shown that 77% of those that had been on sick leave had repeated episodes (6). Even though repeated sick leaves may be related to the base complaint severity and/or chronicity (6, 7), this same study with Brazilian public workers has shown that the main cause of the first sick leave episode changed on subsequent episodes in 55% of the workers that had repeated sick leaves (6). It is important to note that repeated sick leave episodes may lead to long-term sick leaves (8).

Long-term sick leave has already been recognized as a major public health problem, after all it affects the workers as an individual, since it implies not only higher health risks but also increased social and economic vulnerability (9), and the society as a whole, due to loss of valuable workforce and disability benefits (9, 10). Once on disability benefits, there is an increased tendency that people will not go back to work; they rather progress to another benefit or go into disability retirement (10). Thus, facilitating an adequate return to work when facing long-term sickness absence should be a main goal for employers and the society. However, returning to work after long-term sick leaves is a complex, multifactorial, and difficult process (11–13), especially if it involves the so-called readapted return to work. This is a process that aims to facilitate workers (with physical and/or mental conditions) overcoming barriers to return to work. It implies some changes in work content or job and, frequently, in status; these changes may vary depending on the workers' limitations and/or sequelae. It should be highlighted that, if the workers present a good functional recovery they return to work without readaptation; however, a previous study with Brazilian public workers on sick leave has shown that 26% of 956 individuals returned to work only after passing through the readaptation process (14).

The already complex nature of the return-to-work process seems to be even more difficult when workers are on sick leave due to mental health conditions (6, 8, 15). It has already been observed that workers presenting mental illnesses have to deal with many barriers (such as exhaustion, reduced concentration, perfectionism, lack of social support form colleagues and/or supervisor, lack of organizational structures complicating the implementation of work accommodations) that can undermine the process of returning to work (16).

In view of the above considerations, the objectives of this study was to verify whether (a) changes in the classification of the main condition responsible for the first sick leave episode throughout medical examinations and if (b) suffering from mental health illnesses (chapter V of the International Classification of Diseases—ICD-10) were associated with returning to work with readaptation among workers of a Brazilian public university who had repeated episodes of sick leave due to non-work-related injuries and illnesses.

## 2. Materials and methods

### 2.1. Design and setting

A historical cohort study was carried out with non-work-related illnesses suffered by statutory workers of university campuses in a medium-sized city in the state of São Paulo, Brazil, between January 2010 and December 2015.

### 2.2. Ethical aspects

The study was authorized by the Human Resources managers of the five participating institutions (four university units and one administrative unit). It was approved by the Botucatu Medical School Ethics Board (# 1874625, 19 December 2016) in accordance with Brazilian legislation on research involving human subjects (CEP/CONEP Resolution #196/96).

### 2.3. Data

An analysis was conducted on all medical reports from the institutions' own occupational medical service, which examines all statutory workers who request a sick leave with a duration of 2 days or more. This service, which manages all sick leaves and has the power to grant or deny them, is also responsible for the medical boards that decide on return-to-work cases.

This resulted in 5,549 instances of sick leaves among 738 workers. For this analysis, the follow-up included information up to the 20^th^ sick leave of the worker, since 91.8% of workers had up to this number of cases. No worker in the series had changes in the main condition for the sick leave (based on chapter of ICD-10) after the 20^th^ episode.

Data were extracted from two institutional databases: (a) the Integrated Occupational Management Software, which identifies each worker and records medical examinations data; and (b) the Medical Care System, which records data about their return to work.

The first exposure variable of interest was the presence of changes in the ICD-10 chapter regarding the main condition responsible for the first sick leave episode throughout the different medical examinations (dichotomous, presence, or not of a change); the second exposure was having had at least one sick leave episode due to mental illness (dichotomous, having at least one diagnosis in chapter 5 or not), with or without a change in the ICD-10 chapter over time. The (dichotomous) outcome was returning to work with readaptation. The covariates included in the analysis were sex (dichotomous); marital status (dichotomous); being a health professional (dichotomous); the university unit where they worked until readaptation (five categories); age at the beginning of the sick leave process (in years); total time working at the university (in years); and the total time away during the period (in days).

### 2.4. Statistical methods

To achieve the study's objectives we estimated the associations between the exposures and returning to work with readaptation by multiple logistic regression (MLR) and targeted maximum likelihood estimation (TMLE) (17, 18). The use of both methods allowed us not only obtaining odds ratio (OR) measurements and their respective confidence intervals, but also identifying differences in performance that may support the choice of a more advanced computational method (TMLE) compared to a traditionally used one (MLR) to quantify the frequency and impact of workers' health on sick leaves.

Logistic regression (LR) is among the most used techniques to estimate associations. This method allows to interpret the regression coefficients' model in terms of OR. However, depending on the number of units of analysis and independent variables included in a model, its estimates might be biased or not converging (19). Furthermore, LR and other parametric regression models are often incorrectly specified (20).

Targeted maximum likelihood estimation (TMLE) (20, 21) is a recently developed approach to estimating associations, which prevents the occurrence of some of those problems with LR models. It is a method based on maximum likelihood, which can be used to estimate causal effects in observational studies. This is a doubly robust method in that the estimation of a causal effect will not be biased even if the models adopted to describe the expected value of the outcome as a function of the exposure and confounders and the propensity of the exposure as a function of the above have been incorrectly specified, provided that at least the exposure mechanism has been consistently estimated (17). Thus, TMLE has become an attractive alternative to parametric statistical models in applications where the risk of incorrect specification in the data generating distribution increases as the size and complexity of the data set increase (17, 19).

**Figure 1:**
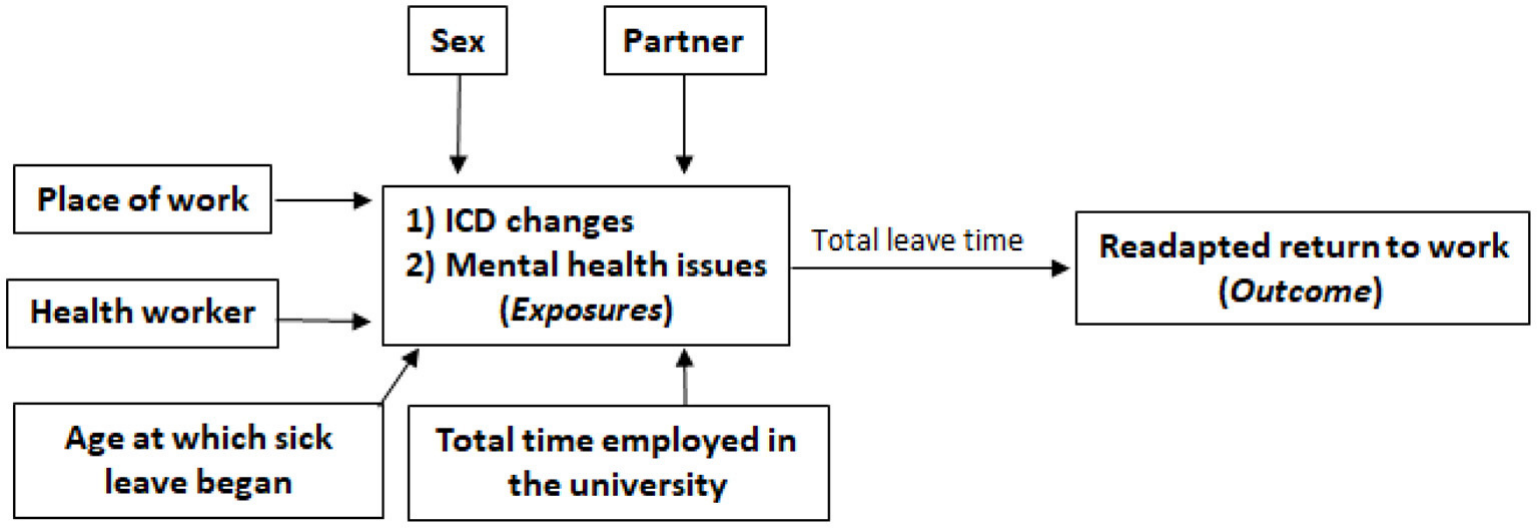
Inferential model to be adjusted by LR and TMLE.

The explanatory models were built *a priori* (Figure 1) according to statistically significant and plausible findings of previous studies that explored this cohort (7, 14). The chances of return with readaptation because of an ICD-10 chapter change and due to the occurrence of at least one illness included in chapter V of the ICD-10 (mental and behavioral disorders) were estimated via MLR, considering the confounding effects of sex, marital status, age of admission, age at start of sick leaves, and the university unit where they worked (place of work). The results obtained with the described parametric models (MLR) were contrasted with the OR estimates obtained with the TMLE method, assuming logistic models to describe the expected value of the outcomes as a function of exposure and confounders. It is noteworthy that the MLR models are accompanied by the estimators for all tested variables, while the TMLE model only has the estimates of the global adjustment of the model, even though they considered the same covariates dynamics in the adjustments.

Finally, the associations obtained by the different models (MLR and TMLE) were compared based on an overlapping analysis of the confidence intervals obtained by both methods for the OR estimation. This decision was based on the belief that LRs have a lower performance, compared to TMLE. It has already been shown, using simulated data that the different versions of TMLE provide more accurate OR estimates when compared to estimates obtained by LR (22). All analyses were performed using R statistical package version 4.0.3.

## 3. Results

The descriptive and comparative results of the sociodemographic and work-related variables are presented in Table 1. Among readapted workers, the high proportion of health workers should be highlighted, as well as the low proportion of ICD-10 chapter changes and the higher occurrence of mental health issues diagnoses. This group of readapted workers had worked at the university for a shorter period and remained away from work for longer.

**Table 1:** Exploratory data analysis between the group of workers who returned to work without readaptation (NR) and those who returned after passing through readaptation (R).

		**NR**		**R**		*p*-value^*^
		***n* (511)**	**%**	***n* (227)**	**%**	
Sex	Female	319	62.4	153	67.4	
	Male	192	37.6	74	32.6	0.194
Presence of partner	No	223	43.6	110	48.5	
	Yes	288	56.4	117	51.5	0.225
Work unit	General administration	24	4.7	5	2.2	
	Agricultural sciences	31	6.1	11	4.8	
	Human health	397	77.7	196	86.3	
	Animal health	27	6.1	9	4.8	
	Biological sciences	32	6.3	6	2.6	0.063
Type of work	Health worker	216	42.3	146	64.3	
	Other	295	57.7	81	35.7	< 0.001
ICD chapter changes in the follow-up	No	1,689	58.5	1,754	65.9	
	Yes	1,200	41.5	906	34.1	< 0.001
Presence of mental health codes (ICD V) in the follow-up	No	326	63.8	87	38.3	
	Yes	185	36.2	140	61.7	< 0.001
		**Median**	**IQR**	**Median**	**IQR**	***p*** **-value** ^**^
Age at start of sick leave process		50.30	45.3–54.6	49.29	45.4–54.5	0.371
Time working at the university (in years)		22.24	16.9–26.1	19.14	17.2–24.9	0.014
Total time absent (in days)	64	26–215		438	130–1,180	< 0.001

* Chi-square test.

** Mann-Whitney U-test.

Table 2 shows the results of the adjustments of the predictive models by both methods for changes in the ICD-10 chapter and returning to work with readaptation. For this exposure, both models were statistically significant as regards protecting the worker for returning to work with readaptation, that is, having had a change in the type of illness during the sick leave process results in a lower chance of returning to work in another function, estimated at 34% by the TMLE model and at 36% by the LR model.

**Table 2:** Predictive models for changes in the ICD-10 chapter and returning to work with readaptation.

		**CI 95% (OR)**		
	***OR***	***L***	***U***	***p*-value**
**TMLE**				
ICD chapter changes in the follow-up (ref. no)	0.66	0.40	0.93	< 0.001
**MLR**				
ICD chapter changes in the follow-up (ref. no)	0.64	0.42	0.97	0.036
Sex (ref. female)	1.23	0.83	1.81	0.303
Marital status (ref. no)	0.84	0.60	1.17	0.312
Type of work (ref. other)	2.56	1.73	3.79	< 0.001
Time working at the university (in years)	1.03	1.00	1.06	0.072
Age at start of sick leave process (in years)	0.99	0.96	1.01	0.341
Work unit (ref. Biological sciences)				0.753
Animal health	1.77	0.55	5.68	0.338
General administration	0.96	0.26	3.56	0.946
Agricultural sciences	1.59	0.51	5.00	0.424
Human health	1.56	0.61	3.98	0.355

Table 3 presents the results of the adjustments of predictive models with both methods for having had at least one diagnosis of an ICD-10 chapter V disease and returning to work with readaptation. For this exposure, both models were statistically significant in increasing the risk of returning to work with readaptation, that is, having had some main diagnosis of a mental health issue results in a significant increase in the chance of returning to work in another role: 2.74 times in the TMLE model and 2.61 times in the LR model.

**Table 3:** Predictive models for the diagnosis of diseases included in chapter V of ICD-10 and returning to work with readaptation.

		**CI 95% (OR)**		
	***OR***	***L***	***U***	***p*-value**
**TMLE**				
Mental health codes (ICD V) involved in the follow-up (ref. no)	2.46	1.66	3.27	< 0.001
**MLR**				
Mental health codes (ICD V) involved in the follow-up (ref. no)	2.61	1.86	3.66	< 0.001
Sex (ref. female)	1.41	0.94	2.09	0.095
Marital status (ref. no)	0.89	0.64	1.26	0.516
Type of work (ref. other)	2.33	1.56	3.46	< 0.001
Time working at the university (in years)	1.03	1.00	1.06	0.055
Age at start of sick leave process (in years)	0.99	0.96	1.02	0.495
Work unit (ref. Biological sciences)				0.762
Animal health	1.62	0.50	5.27	0.425
General administration	0.83	0.22	3.14	0.782
Agricultural sciences	1.48	0.47	4.68	0.506
Human health	1.46	0.57	3.76	0.435

When comparing the results obtained by the MLR and TMLE techniques in Table 4, it can be observed that a relatively high rate of overlapping between the 95% confidence intervals for the return to work with readaptation OR occurs due to both the “change in the ICD-10 chapter” exposure and “having had at least one ICD-10 chapter V diagnosis” exposure.

**Table 4:** Comparison of predictive models for the exposures regarding ICD-10 chapter changes and diseases included in ICD-10 chapter V adjusted for the covariates and the return to work with readaptation outcome.

**Exposure**	**Methodology**		**LR/TMLE variation (%)**
	**Adjusted by LR**	**Programmed TMLE**	
	***OR*** **(*****CI*** **95%)**	***OR*** **(*****CI*** **95%)**	
ICD-10 chapter changes	0.65 (0.43–0.98)	0.66 (0.40–0.93)	−1.53 (7.5–5.3)
ICD-10 chapter V	2.61 (1.86–3.66)	2.46 (1.66–3.27)	7.41 (12.04–11.92)

## 4. Discussion

Among the 738 public workers who had had two or more sick leaves and who generated 5,549 instances of sick leaves during the study period, it was found that 227 of them returned to work in different activities and/or roles from those they performed before the beginning of their illness process; a return that occurred, for the most part (91%), in up to 20 different episodes of work absenteeism due to a sick leave.

As far as the authors know, this is the first study to verify the two exposures under study. Regardless of the analytical model, the findings show that having had a change in the ICD-10 chapter regarding the main condition decreases the likelihood of returning to work in another role by over 30%. On the other hand, having had at least one sick leave due to a cause included in chapter V of the ICD-10 (mental and behavioral disorders) more than doubles the chance of not returning to work in the same activity that was being performed before. A previous study with this same group of workers, but also including those who had only one episode of sick leave, has shown that among the mental health conditions depressive disorders and the use of psychoactive substances were the most frequently documented as the main cause of sick leaves (6).

Even though health problems that are serious enough to prevent the subject from working are not natural, expected, or desirable, our results show that some health events seem to attenuate or aggravate their evolution. Therefore, reaching workers early and following them up during their sick leave process, to support the rehabilitation through adaptation of return to work interventions to each individuals' unique situation and needs, may be essential to prevent loss of work capacity and changes in work content or job after returning to work (23). This reinforces the need to seek more evidence that can support the follow-up of workers in processes of illness to interfere in their course, since the impacts caused by returning to work, especially in different roles and/or places to the ones before the illness, extend far beyond the sphere of the job itself, worsening the individual's health prognosis, particularly in relation to mental health, as shown by the findings of this study.

Our results also show that it is necessary to optimize return to work interventions, especially those aimed at workers with mental health problems. Even though this study did not aim to evaluate the effectiveness of return to work interventions, it is important to bring to the attention of the readers the results of a recent systematic review which has demonstrated that implementing work-focused cognitive behavioral therapy interventions helps reduce lost time and costs associated with work disability for workers on sick leave due to mental health conditions (24). Also, it has already been shown that the incorporation of a multi-domain (encompassing health-focused, service coordination, and work modification interventions) early return to work program, that adopts a biopsychosocial model of care, with the involvement of a return to work coordinator increases return to work success (24–26), since it addresses a great deal of factors that impact return to work (24).

Although some inferences can be drawn about the predictors of returning to work with a change of role or activity, the analyses developed here were not intended as a broad investigation of these variables, since this topic had already been developed in more depth (6, 7, 14) by the authors with all the variables offered by the database. However, to test whether different statistical models could produce different estimators and, thus, point to alternative methodological options in situations of testing for repeated measures with different numbers of repetitions across subjects (a situation in which the performance of more traditional tests, such as logistic regressions, is greatly compromised), a plausible theoretical causal model was developed from the previous findings (which included few variables) and analyzed using a TMLE model.

Targeted maximum likelihood estimation is a more robust technique, compared to logistic regression models (17, 20, 22), given that the outcomes studied are dichotomous. However, its higher predictive performance is widely known in randomized clinical trials, which is not the design of this research. Therefore, the scarcity of literature on its use in observational studies with predictive capacity (as in the case of a historical cohort) can be seen as both a positive point and a limitation. Since TMLE is used to analyze censored observational data from a non-controlled experiment in a way that allows effect estimation even in the presence of confounding factors, it seemed a reasonable choice as an alternative approach to the analysis.

Thus, after running both models, the TMLE results did not differ from the MLR results, either by promoting no changes in the statistical significance of the associations between the exposures and the outcome (although possible, it was not expected) or in the magnitude of the associations (more probable and expected, in light of the literature). This is why it was not possible to state whether one model behaves more conservatively than the other, even for a large database such as the one studied here.

The comparison between the MLR and TMLE techniques by overlapping the confidence intervals considered the sampling variability, as this proved to be more appropriate than comparing the results from the simple division between the OR obtained by the two techniques, even though both were presented. The relatively high proportion of overlapping between the confidence intervals obtained suggests that, in this sample, the analytical models did not present differences in terms of understanding the probability of returning to work with readaptation due to changes in the ICD-10 chapter or having had at least one disease in chapter V, which does not allow ruling out the possibility of TMLE offering advantages in other databases with other dynamics between the variables.

Due to the difficulties that TMLE has in its execution, first because it is little known, then because it is unavailable on platforms other than R, and finally, because its process is less illustrative and understandable than that of LR, there was no advantage to using the method in this case. Regarding the understanding of the results, which seems to be the most important point for an epidemiological interpretation, i.e., the perspective of this study, it is worth mentioning that, when using TMLE, an *a priori* causal theoretical model that is plausible to the researcher must be developed. However, when using LR, the fact that it can establish adjustments of simple regression models with entry and exit criteria in the multiple model (which may be even less conservative) as a primary step means that the model can be structured from the results obtained for the predictive variables individually, without assuming a causal model. We consider this assumption of a causal model before understanding the influence of the variables on the outcome to be arbitrary. However, in a scenario of high incidence and large OR, such as the one found here, where the adjusted OR could be more adequate, the adjustment techniques tend to exaggerate the associations (27). Thus, the use of TMLE can be an interesting alternative for smoothing the associations.

Furthermore, this intermediate step taken by the simple models allows visualizing the behavior of the associations, even in the sense of perceiving the existence of collinearities between predictive variables and also suggesting potential confounding variables in the association between exposure and outcomes.

The findings of this investigation are not able to confirm the previous published literature on the benefits of TMLE. However, it is very important to examine some alternative methods for predictive analyses to provide more accurate estimators of associations, especially in complex research scenarios. Since its use is not yet among the first choices and because the application literature exists but is not extensive, it is not possible to discard its benefits. More applications and analyses are thus expected in different architectures and, mainly, model comparisons, so that there are always more options for analysis.

## 5. Conclusions

Changes in the classification of the main condition between repeated medical examinations and diseases related to mental health were associated with returning to work with readaptation in workers of a Brazilian public university, in the sense that a history of changes in the ICD-10 chapters reduces the chances of the outcome, and that mental health conditions more than double its chance of occurrence, regardless of the estimation method used. This allows concluding that there were no advantages in the use of TMLE, given the difficulties in access, use, and the assumptions of the technique.

## Data availability statement

The raw data supporting the conclusions of this article will be made available by the authors, without undue reservation.

## Ethics statement

The studies involving human participants were reviewed and approved by # 1874625, 19 December 2016. The patients/participants provided their written informed consent to participate in this study.

## Author contributions

AD, HN, CR-F, JG-S, JB, and JL-R: conceptualization and writing—review and editing. AD, MS, JB, and JG-I: data curation. AD, HN, CR-F, and JB: formal analysis. AD, HN, MS, and JB: investigation. CR-F, JG-S, JB, JG-I, and JL-R: methodology. CR-F, JG-S, JG-I, and JL-R: project administration. AD, HN, CR-F, JG-S, MS, JB, JG-I, and JL-R: resources. AD, CR-F, MS, and JL-R: software. CR-F, JG-S, JB, and JL-R: supervision. HN, CR-F, JG-S, JG-I, and JL-R: validation. AD, HN, JG-S, JB, JG-I, and JL-R: visualization. AD, HN, MS, JB, JG-I, and JL-R: writing—original draft. All authors contributed to the article and approved the submitted version.
